# Keratinocyte‐derived IL‐1β induces *PPARG* downregulation and *PPARD* upregulation in human reconstructed epidermis following barrier impairment

**DOI:** 10.1111/exd.14323

**Published:** 2021-03-18

**Authors:** Stefan Blunder, Thomas Krimbacher, Verena Moosbrugger‐Martinz, Robert Gruber, Matthias Schmuth, Sandrine Dubrac

**Affiliations:** ^1^ Department of Dermatology, Venereology and Allergology Medical University of Innsbruck Innsbruck Austria

**Keywords:** epidermal barrier function, human epidermal equivalents, keratinocytes, nuclear hormone receptor, PPAR

## Abstract

Peroxisome proliferator‐activated receptors (PPARs) are a family of nuclear hormone receptors. In skin, PPARs modulate inflammation, lipid synthesis, keratinocyte differentiation and proliferation and thus are important for skin barrier homeostasis. Accordingly, PPAR expression is altered in various skin conditions that entail epidermal barrier impairment, that is atopic dermatitis (AD) and psoriasis. Using human epidermal equivalents (HEEs), we established models of acute epidermal barrier impairment devoid of immune cells. We assessed PPAR and cytokine expression after barrier perturbation and examined effects of keratinocyte‐derived cytokines on PPAR expression. We show that acetone or SDS treatment causes graded impairment of epidermal barrier function. Furthermore, we demonstrate that besides IL‐1β and TNFα, IL‐33 and TSLP are highly relevant markers for acute epidermal barrier impairment. Both SDS‐ and acetone‐mediated epidermal barrier impairment reduce *PPARG* expression levels, whereas only SDS enhances *PPARD* expression. In line with findings in IL‐1β and TNFα‐treated HEEs, abrogation of IL‐1 signalling restores *PPARG* expression and limits the increase of *PPARD* expression in SDS‐induced epidermal barrier impairment. Thus, following epidermal barrier perturbation, keratinocyte‐derived IL‐1β and partly TNFα modulate *PPARG* and *PPARD* expression. These results emphasize a role for PPARγ and PPARβ/δ in acute epidermal barrier impairment with possible implications for diseases such as AD and psoriasis.

**Abbreviations**
AD: atopic dermatitis
DAMP: damage‐associated molecular pattern
HEEs: human epidermal equivalents
IL‐1α: Interleukin‐1 alpha
IL‐1β: Interleukin‐1 beta
IL‐33: Interleukin 33
LY: Lucifer yellow
SC: stratum corneum
SDS: sodium dodecyl sulphate
TEER: transepithelial electrical resistance
TSLP: thymic stromal lymphopoietin

## 1 INTRODUCTION

Peroxisome proliferator‐activated receptors (PPARs) are a family of ligand‐dependent nuclear hormone receptors that comprise PPARα, PPARβ/δ and PPARγ. After ligand activation, PPARs heterodimerize with the retinoid‐X‐receptor (RXR). Together with co‐factors the complex binds to PPAR response elements (PPRE) in promoter regions of target genes and regulates gene expression. PPARs are activated by numerous endogenous and exogenous fatty acids and derivatives.^1^ PPARα and PPARγ are primarily expressed in the suprabasal layers of the epidermis, whereas PPARβ/δ is present throughout the entire epidermis.^1^, ^2^ In skin, PPARs modulate inflammation, lipid synthesis, keratinocyte differentiation and proliferation.^1^, ^2^, ^3^, ^4^, ^5^, ^6^ Furthermore, PPARs are involved in the homeostasis of skin appendages including hair follicles and sebaceous glands.^5^, ^7^, ^8^ Activation of all three PPARs improves barrier recovery after acute disruption.^1^, ^2^, ^3^, ^4^, ^9^ Moreover, PPAR expression is modified in various inflammatory skin conditions that entail epidermal barrier impairment. Depending on the experimental set‐up, PPARα expression is decreased or remains unchanged in atopic dermatitis (AD) and psoriasis, respectively.^3^, ^10^, ^11^, ^12^, ^13^ PPARβ/δ has been shown to be upregulated in psoriasis.^10^, ^12^, ^14^, ^15^, ^16^ Although information on PPARγ is contradictory in AD, it was shown to be consistently reduced in psoriasis.^10^, ^12^, ^13^, ^14^, ^17^, ^18^ In a model of acute epidermal barrier impairment, PPARα and PPARγ expression is decreased, whereas PPARβ/δ is not altered in human skin.^19^ In contrast, a more recent study reports that acetone‐mediated barrier perturbation increases *PPARD* and diminishes *PPARA* and *PPARG* expression levels in organotypic skin cultures.^20^ In agreement with expression data, PPARβ/δ activation induces psoriasis‐like skin symptoms, which were shown to be ameliorated by PPARβ/δ antagonism.^14^, ^21^, ^22^ Furthermore, PPARγ activation was shown to ameliorate skin lesions in patients with psoriasis.^4^, ^23^, ^24^, ^25^ Accordingly, a novel and specific PPARγ modulator was proven to be anti‐inflammatory and anti‐proliferative and to restore differentiation in a psoriasis‐like mouse model.^26^ Together, these data exemplify the diverse role of PPARs in modulating skin inflammation and underscore their importance in skin barrier homeostasis. Thus, in the present study we established reproducible models of epidermal barrier impairment closely mimicking human skin by using human epidermal equivalents (HEE). In these models, we assessed cytokine and PPAR alterations at gene expression level after epidermal barrier impairment and evaluated the modulatory impact of cytokine changes on PPARs.

## 2 METHODS

### 2.1 Keratinocyte isolation

The study was approved by the Ethics Committee of the Medical University of Innsbruck and conducted in accordance with the Declaration of Helsinki principles. All study subjects gave written informed consent and participated voluntarily.

Primary keratinocytes were isolated from non‐UV‐irradiated trunk skin of nine subjects undergoing plastic surgery. After digestion with a mixture of Trypsin/Dispase (2:1) (SigmaAldrich, St. Louis, MO) at 4°C for 16–20 h, keratinocytes were cultured in CellnTec basal media (CnT‐BM.1, Bern, Switzerland) supplemented with CellnTec human keratinocyte growth supplement (CnT‐07.S). The medium was changed every other day. At 70–80% confluency, cells were collected and then stored until further use. For generation of human epidermal equivalents (HEEs), second passage keratinocytes were used.

### 2.2 Generation of human epidermal equivalents (HEEs)

Human epidermal equivalents were generated as described previously.^27^ In short, keratinocytes were harvested by trypsinization, pelleted and seeded at a density of 3.4 × 10^5^ on 0.4‐µm inserts (Merck Millipore, Billerica, MA) in CellnTec growing medium. After 2 days of submerged culturing, media were switched to calcium‐chloride enriched CnT‐02‐3D medium (CellnTec, Bern, Switzerland). 16 h later, HEEs were lifted to the air‐liquid interface by aspiration of the medium inside the inserts. Thereafter, culture media were changed daily until harvesting. HEEs were grown at a humidity of 50–60%, at 37°C and 5% CO_2_.

### 2.3 Barrier disruption in HEEs

The stratum corneum (SC) of HEEs was exposed to 1% Sodium Dodecyl Sulphate (SDS; SigmaAldrich, St. Louis, MO) or vehicle control (PBS) for 1 min or to acetone (SigmaAldrich, St. Louis, MO) or vehicle control (PBS) for 5 min.

### 2.4 Keratinocytes and HEE treatment

Keratinocyte monolayer cultures and HEEs were stimulated with human IL‐1β (c: 100 ng/ml), TNFα (c: 10 ng/ml) **(**both CellGro, Corning, NY) or TSLP (c: 10 ng/ml) (R&D systems, Minneapolis, MN) for 6 h or 24 h. Anakinra (Kineret, r‐metHuIL‐1ra, BoehringerIngelheim, Vienna, Austria) at a concentration of 50 µg/ml or Infliximab (Remicade, JanssenBiotech, Leiden, Netherlands) at a concentration of 100 µg/ml were added to the medium of HEEs 24 h and right before SDS application.

### 2.5 Morphological analysis

Human epidermal equivalents were fixed in 4% formaldehyde, paraffin‐embedded and 6 µm sections were stained with haematoxylin & eosin. Sections were analysed using an Olympus BH‐2 light microscope (Olympus, Shinjuku, Japan) equipped with a ProgRes C10plus camera (Jenoptik, Jena, Germany) and ProGresCapturePro 2.8.8 image analysis software (Jenoptik, Jena, Germany).

### 2.6 Lucifer yellow permeability assay

200 µl of 1 mM Lucifer Yellow (SigmaAldrich, St. Louis, USA) was applied onto HEEs and incubated for 2 h at 37°C. Then, HEEs were rinsed with PBS, fixed in formaldehyde and paraffin‐embedded. Finally, 6 µm deparaffinized sections were counterstained with DAPI and inspected in an Olympus BX60 epifluorescence microscope (Olympus, Shinjuku, Japan).

### 2.7 Transepithelial electrical resistance (TEER) measurements

Transepithelial electrical resistance measurements were performed using an Epithelial Volthommeter (World Precision Instruments, Sarasota, FL) according to manufacturer's instructions. Measurements were recorded using fresh 0.5 ml of CnT‐02‐03D medium on top of the transwell and 1 ml below the transwell.

### 2.8 RNA Isolation and RT‐ PCR

Total RNA from cultured keratinocytes and HEEs was isolated using TRIZOL reagent (Gibco BRl, Life Technology). DNA‐free kit (Ambion, Carlsbad, CA) was used to remove contaminating gDNA from RNA preparation according to the manufacture's protocol. RNA integrity was evaluated by agarose gel electrophoresis and RNA quantity was determined by spectrophotometry. Thereafter, 1 µg RNA was reverse transcribed using Superscript II RNase H‐reverse transcriptase (Life Technologies, Vienna, Austria). Levels of gene expression were analysed by quantitative PCR using the Biorad CFX 96 real‐time PCR detection system and the TaqMan Brilliant III Ultrafast Quantitative PCR MasterMix Kit from Agilent Technologies (Santa Clara, CA, USA). TaqMan^®^ Gene Expression Assays for most genes used were purchased from Applied Biosystems (Foster City, CA). Primers and Probes specific for human TATA‐binding protein were synthesized by Microsynth (Balgach, Switzerland) and selected by Primer Express software (Applied Biosystems, Foster City, CA). To control for variations in RNA quantity, gene of interest (GOI) expression levels were normalized to the expression of TATA box binding protein. Relative expression levels were calculated using the 2^−ΔΔCT^ method.

### 2.9 Statistical analysis

Statistical analyses were performed using GraphPad Prism 6 software (GraphPad Software, LaJolla, CA). Data are presented, if not other specified, as mean ± SEM. Statistical significance as determined using Student's paired two‐tailed t test or one‐way analysis of variance, followed by Bonferroni post hoc test was performed with significance determined as a *p*‐value <0.05.

## 3 RESULTS

### 3.1 SDS perturbs epidermal barrier function in HEEs more efficiently than acetone

**FIGURE 1:**
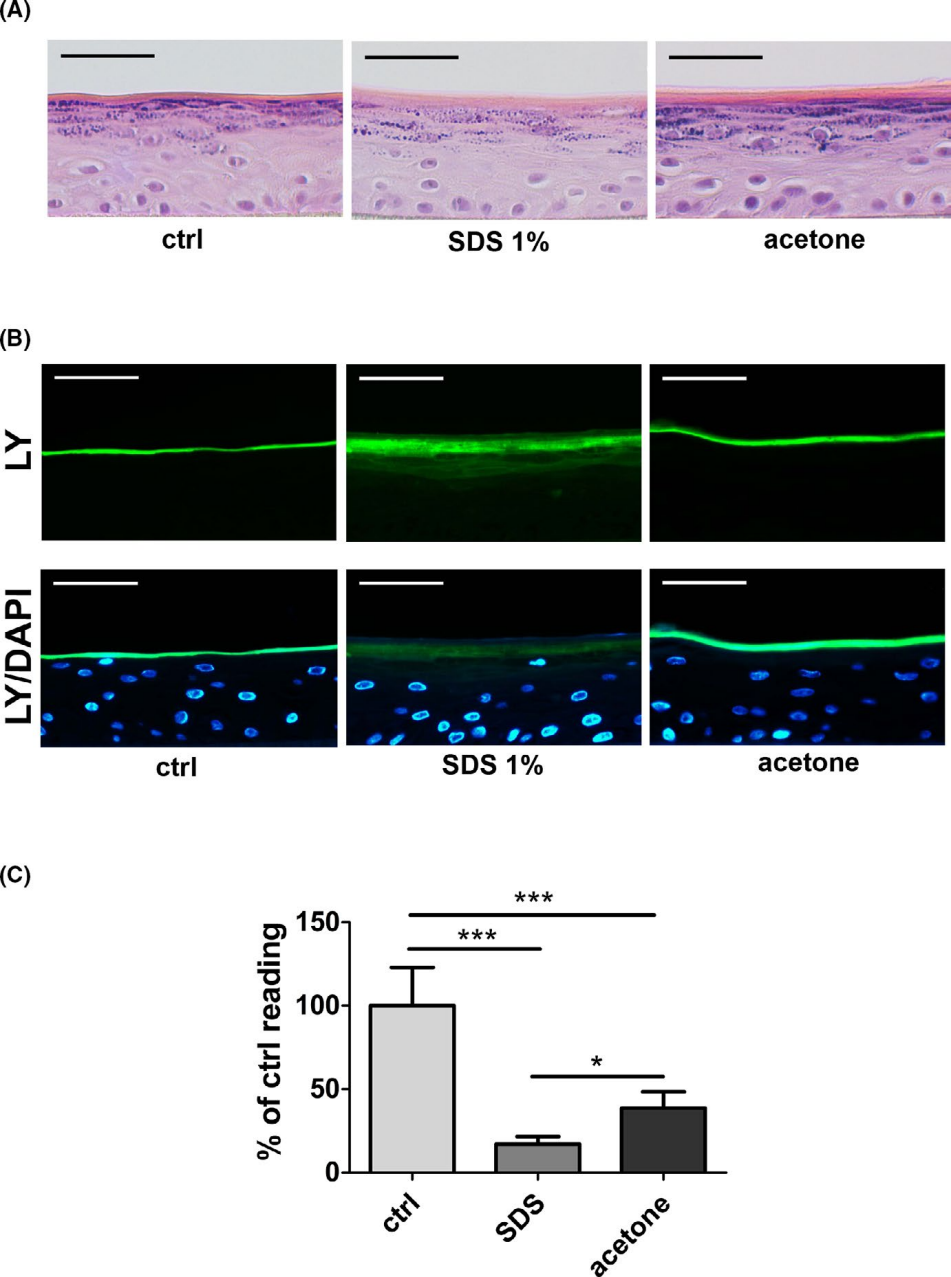
Sodium dodecyl sulphate‐ and acetone‐mediated barrier impairment in HEEs. (A) Representative H&E staining of PBS‐ (ctrl), SDS‐ and acetone‐treated HEEs after treatment. Bars = 50 µm. (B) Representative images from Lucifer Yellow (LY)‐treated HEEs after PBS‐ (ctrl), SDS‐ or acetone‐treatment. DAPI show nuclei. 3–4 independent experiments were carried out. Bars = 50 µm. (C) TEER measurement of PBS‐ (ctrl), SDS‐ and acetone‐treated HEEs. Values are given as % of ctrl group measurements. Combined data of 2 independent experiments are shown, *n* = 7. Data are presented as mean ± SEM. One‐way analysis of variance, followed by Bonferroni post hoc test was performed. *p = <0.05; ***p = <0.001. LY, lucifer yellow; TEER, transepithelial electrical resistance; SDS, sodium dodecyl sulphate

First, we established an *in vitro* model to study epidermal barrier impairment. We generated human epidermal equivalents (HEEs) from primary human keratinocytes cultured at a humidity of 50–60% that closely mimic human epidermis.^27^, ^28^ On day 12 of culture, we exposed HEEs to 1% sodium dodecyl sulphate (SDS) or acetone, two agents commonly used to inflict barrier perturbation in skin.^19^, ^29^ Haematoxylin & eosin (H&E) staining proves that SDS and acetone do not have toxic effects on the integrity of HEEs (Figure 1A). To assess whether SDS or acetone perturbs epidermal barrier function, we (i) employed Lucifer Yellow (LY) permeability assay and (ii) measured transepithelial electrical resistance (TEER), two techniques used to evaluate the outside‐in (mainly the stratum corneum) and inside‐out (tight junctions and stratum corneum) barrier competence, respectively. While SDS, but not acetone, rendered HEEs permeable to LY (Figure 1B), both SDS and acetone induced a striking decrease of TEER, indicating barrier impairment (Figure 1C). These data show that both SDS and acetone application successfully perturbs epidermal barrier function, yet to a different degree. Indeed, TEER is a highly sensitive method reflecting the ionic conductance of the paracellular pathway in HEEs. LY assay is less sensitive than TEER measurements due to the size of the molecule (C_13_H_10_Li_2_N_4_O_9_S_2_).^30^ Thus, our results show that acetone only mildly disrupts the epidermal barrier, whereas SDS has a more deleterious effect.

### 3.2 In HEE SDS‐mediated—not acetone‐mediated—barrier perturbation leads to changes of cytokines implicated in AD and psoriasis

**FIGURE 2:**
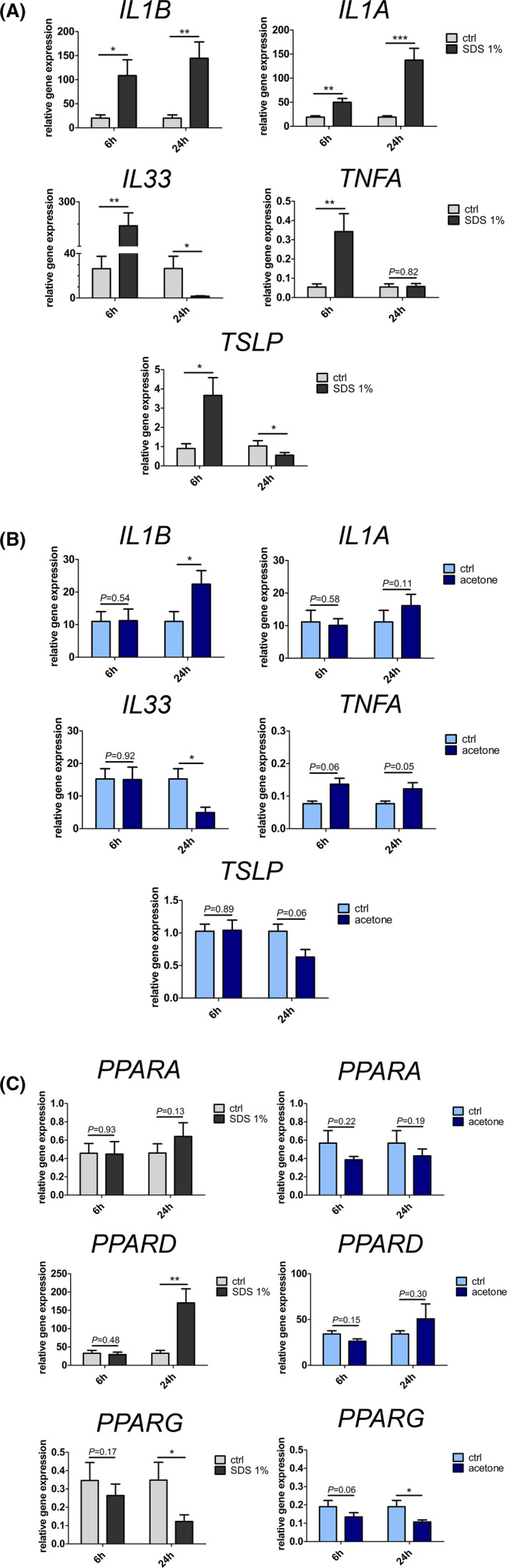
Cytokine and PPAR expression in HEEs with impaired barrier function. (A) HEEs were exposed to SDS 1% and harvested 6 h and 24 h later. mRNA expression levels of cytokines were assessed by RT‐PCR. Combined data from 9 independent experiments are presented as mean ± SEM. (B) HEEs were exposed to acetone and harvested 6 h and 24 h later. mRNA expression levels of cytokines were assessed by RT‐PCR. Combined data from 5 independent experiments are presented as mean ± SEM. (C) HEEs were exposed to SDS 1% or acetone and harvested 6 h and 24 h later. mRNA expression levels of *PPARA*, *PPARD* and *PPARG* were assessed by RT‐PCR. Combined data from 9 independent experiments for SDS‐treated HEEs and from 5 independent experiments for acetone‐treated HEEs are presented as mean ± SEM. Data were analysed using a paired Student's *t* test for all experiments. *p = <0.05; **p = <0.01; ***p = <0.001. *IL1B*, interleukin‐1β; *IL1A*, interleukin‐1α; *IL33*, interleukin 33; *TNFA*, tumor necrosis factor α; *TSLP*, thymic stromal lymphopoietin; *PPARA*, peroxisome proliferator‐activated receptor α; *PPARD*, peroxisome proliferator‐activated receptor β/δ; *PPARG*, peroxisome proliferator‐activated receptor γ

Next, we examined mRNA expression levels of inflammatory genes known to be produced by keratinocytes after barrier impairment in SDS‐ or acetone‐exposed HEEs. The expression of *IL1B* and *IL1A* was increased 6 and 24 h after SDS treatment, respectively (Figure 2A). *TNFA* gene expression was enhanced 6 h (11.9‐fold±5.6; *p* = 0.0119) and remained unchanged 24 h post‐SDS‐mediated barrier perturbation. As illustrated in Figure 2A, *IL33* and *TSLP*, mRNA levels were increased 6 h after SDS application (13.9‐fold±5.6, *p* = 0.0090 and 4.3‐fold±0.8 *p* = 0.0104, respectively). Intriguingly, gene expression levels of both cytokines were decreased at 24 h post‐SDS treatment (IL33: 14.1‐fold±3.5, *p* = 0.0495; TSLP: 2.1‐fold±0.3, *p* = 0.0112 Figure 2A). As depicted in Figure 2B, in acetone‐treated HEEs, *IL1B* mRNA levels were only altered 24 h after acetone treatment (+2.6‐fold±0.7, *p* = 0.0201). *TNFA* gene expression was increased at both time points (1.9‐fold±0.9, [6 h], 1.6‐fold 0.6 [24 h]), whereas *IL1A* gene expression levels remained unchanged. Similar to data from SDS‐treated HEEs, *IL33* and *TSLP*, mRNA levels were reduced 24 h after barrier perturbation elicited through acetone (4.7‐fold±2.1, *p* = 0.0393 and 1.9‐fold±0.4, *p* = 0.0613, respectively, Figure 2B). Yet, gene expression levels of both cytokines remained unaltered 6 h after acetone treatment. Taken together, these findings show that an increase of *IL1B* mRNA levels and a downregulation of *IL33* and *TSLP* gene expression levels 24 h postepidermal barrier perturbation occur regardless of the type of barrier disruptor and consequently of the degree of barrier impairment. In contrast, *IL1A* and *TNFA* are only modulated following more pronounced barrier impairment as produced by SDS. Thus, it is tempting to speculate that expression of these inflammatory mediators allows to evaluate the extent of epidermal barrier impairment in various skin disorders.

### 3.3 SDS‐mediated barrier impairment results both in *PPARG* downregulation and *PPARD* upregulation, while acetone‐mediated barrier perturbation only leads to *PPARG* downregulation

Since in other tissues proinflammatory cytokines have been shown to modulate PPAR expression, we next measured the mRNA level of all three PPAR isotypes.^31^, ^32^ Notably, *PPARD* was increased 5.7‐fold (±0.6; *p* = 0.0027) 24 h but not 6 h after SDS exposure. In contrast, barrier impairment due to acetone application did not result in a significant upregulation of *PPARD*. While *PPARG* remained unchanged 6 h post SDS‐mediated barrier abrogation, it was reduced 3.1‐fold (±0.5; *p* = 0.0106) 24 h post SDS‐mediated barrier abrogation. Likewise, in acetone‐treated HEEs, *PPARG* expression was diminished by 1.7‐fold (±0.2; *p* = 0.0260) at 24 h (Figure 2C). Both SDS‐ and acetone‐mediated barrier impairment did not significantly alter *PPARA* expression (Figure 2C). Thus, epidermal barrier perturbation inflicted by SDS treatment recapitulates PPAR gene expression changes in psoriasis.^10^, ^12^, ^14^, ^15^, ^16^ In contrast, acetone‐induced barrier impairment drives *PPARG* downregulation, which has been inconsistently reported in AD.^13^, ^18^ Together, these findings show that *PPARG*, in contrast to *PPARD* and *PPARA*, is consistently downregulated after SDS‐ and acetone‐induced barrier impairment. These data suggest that *PPARG* expression is a highly sensitive marker for the fitness of the epidermal barrier and serves as the PPAR isoform most closely linked to the integrity of the epidermal barrier.

### 3.4 IL‐1β and TNF‐α treatments trigger a SDS‐like cytokine response in HEEs

**FIGURE 3:**
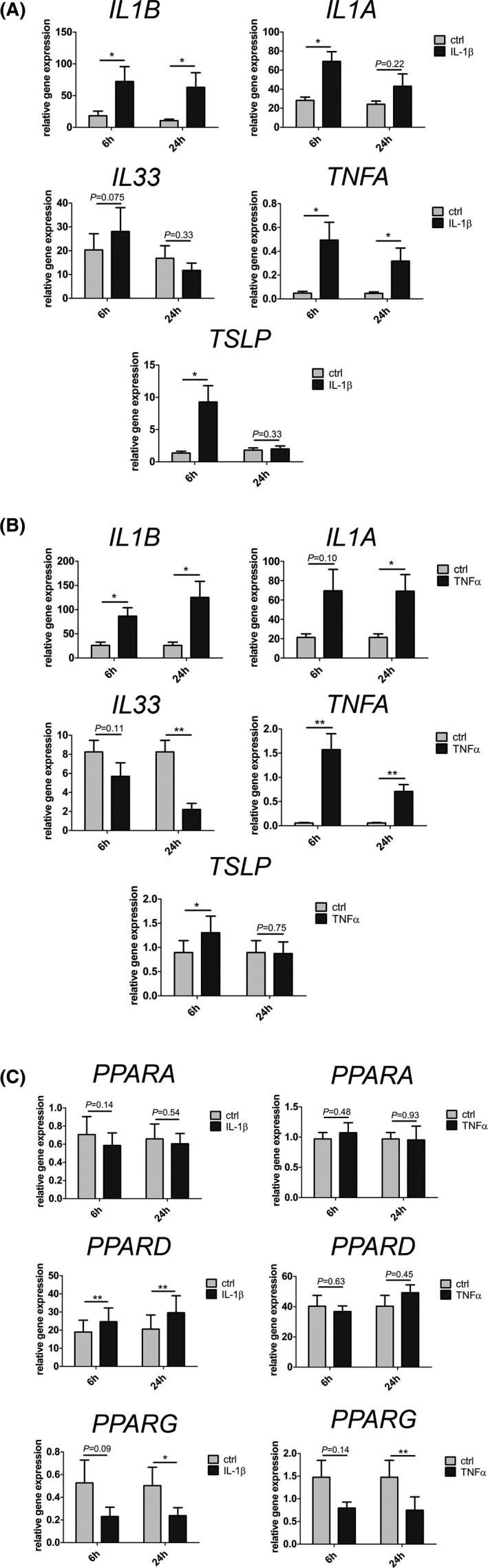
Cytokine and PPAR expression in IL‐1β and TNFα‐treated HEEs. (A) HEEs were treated with IL‐1β for 6 h and 24 h. mRNA expression levels of cytokines were assessed by RT‐PCR. Combined data from 5 independent experiments are presented as mean ± SEM. (B) HEEs were treated with TNF‐α for 6 h and 24 h. mRNA expression levels of cytokines were assessed by RT‐PCR. Combined data from 5 independent experiments are presented as mean ± SEM. (C) HEEs were treated with IL‐1β or TNFα and harvested 6 h and 24 h later. mRNA expression levels of *PPARA*, *PPARD* and *PPARG* were assessed by RT‐PCR. Combined data from 5 independent experiments are presented as mean ± SEM. Data were analysed using a paired Student's *t* test for all experiments. *p = <0.05; **p = <0.01; ***p = <0.001. *IL1B*, interleukin‐1β; IL‐1β, interleukin‐1β; *IL1A*, interleukin‐1α; *IL33*, interleukin 33; *TNFA*, tumor necrosis factor α; TNFα, tumor necrosis factor α; *TSLP*, thymic stromal lymphopoietin; *PPARA*, peroxisome proliferator‐activated receptor α; *PPARD*, peroxisome proliferator‐activated receptor β/δ; *PPARG*, peroxisome proliferator‐activated recpetor γ

Since *IL1B* is upregulated in both SDS‐ and acetone‐mediated barrier impairment, we treated human primary keratinocytes and HEEs with IL‐1β (Figures 3, S1). In HEEs, IL‐1β treatment for 6 h and 24 h led to an increase of *IL1B* and *TNFA* mRNA levels (Figure 3A). *IL1A* and *TSLP* expression levels were upregulated 2.6‐fold (±0.5) and 7.0‐fold (±1.2) 6 h after IL‐1β treatment, respectively, and remained unchanged after 24 h (Figure 3A). *IL33* mRNA was not significantly altered in HEEs treated with IL‐1β (Figure 3A). Similar to IL‐1β, *TNFA* mRNA levels were increased after barrier impairment by either SDS or acetone. TNF‐α treatment for 6 h and 24 h increased *IL1B* and *TNFA* expression (Figure 3B). *IL1A* mRNA was upregulated in HEEs when treated with TNF‐α for 24 h. Similar to IL‐1β treatment, TNF‐α enhanced *TSLP* expression after 6 h. As opposed to IL‐1β treatment, TNF‐α treatment of HEEs for 24 h led to a decrease in *IL33* mRNA (4.9‐fold, ±1.4) (Figure 3B). These results show that IL‐1β and TNF‐α treatments alter *IL1A*, *IL1B*, *TNFA* and *TSLP* expression similar to more pronounced barrier perturbation facilitated by SDS. Moreover, a significant decrease of *IL33* occurs only after TNF‐α treatment.

### 3.5 IL‐1β decreases *PPARG* and increases *PPARD* gene expression in HEEs, whereas TNF‐α only reduces *PPARG* mRNA levels

We next explored in more detail the role of IL‐1β and TNF‐α in primary human keratinocytes and in our HEEs on PPAR expression (Figure 3C and Figure S1). In IL‐1β‐treated primary human keratinocytes, *PPARD* gene expression was increased at 1 h, 3 h and 6 h (1.6‐fold±0.1, 2.4‐fold±0.3, 1.6‐fold±0.2, respectively). In contrast, *PPARG* mRNA levels were decreased from 6 h on (6 h: 1.2‐fold±0.1, 24 h: 1.4‐fold±0.1). Similarly, *PPARA* expression was decreased, yet only slightly, at 6 h (Figure S1). In HEEs, IL‐1β treatment did not impact *PPARA* expression. Yet, paralleling data from primary human keratinocytes, IL‐1β enhanced *PPARD* expression at 6 h (1.4‐fold, ±0.1; *p* = 0.0076) and also 24 h (1.7‐fold, ±0.2; *p* = 0.0047) post treatment start in HEEs (Figure 3C). Notably, IL‐1β treatment reduced *PPARG* expression 2.3‐fold (±0.3; *p* = 0.0942) and 2.2‐fold (±0.3 fold; *p* = 0.0346) 6 h and 24 h post‐treatment, respectively (Figure 3C).

TNF‐α treatment of primary human keratinocytes led to an inconsistent increase of *PPARD* gene expression and a decrease of *PPARG* mRNA levels at 24 h (1.7‐fold±0.1). *PPARA* expression remained unchanged (Figure S1). By contrast, TNF‐α treatment did not alter *PPARA* and *PPARD* mRNA levels in HEEs. *PPARG* expression remained unchanged 6 h, yet it was reduced 1.9‐fold (±0.2; *p* = 0.0260) 24 h post‐TNF‐α treatment of HEEs (Figure 3C).

Overall, data both acquired in primary human keratinocytes and in HEEs largely concur. Yet, minor differences have been observed, likely resulting from the distinct nature of the model systems (keratinocyte monolayer vs. stratified epidermis). These findings demonstrate that both IL‐1β and TNF‐α treatment results in *PPARG* downregulation in HEEs. Furthermore, IL‐1β but not TNF‐α treatment increased *PPARD* expression in HEEs. Thus, these data confirm that IL‐1β treatment mimics the effects of SDS‐mediated barrier perturbation in HEEs.

### 3.6 Blocking of IL‐1 receptor signalling yet not of TNF‐α signalling reverses SDS‐induced changes in *PPARD* and *PPARG* expression

**FIGURE 4:**
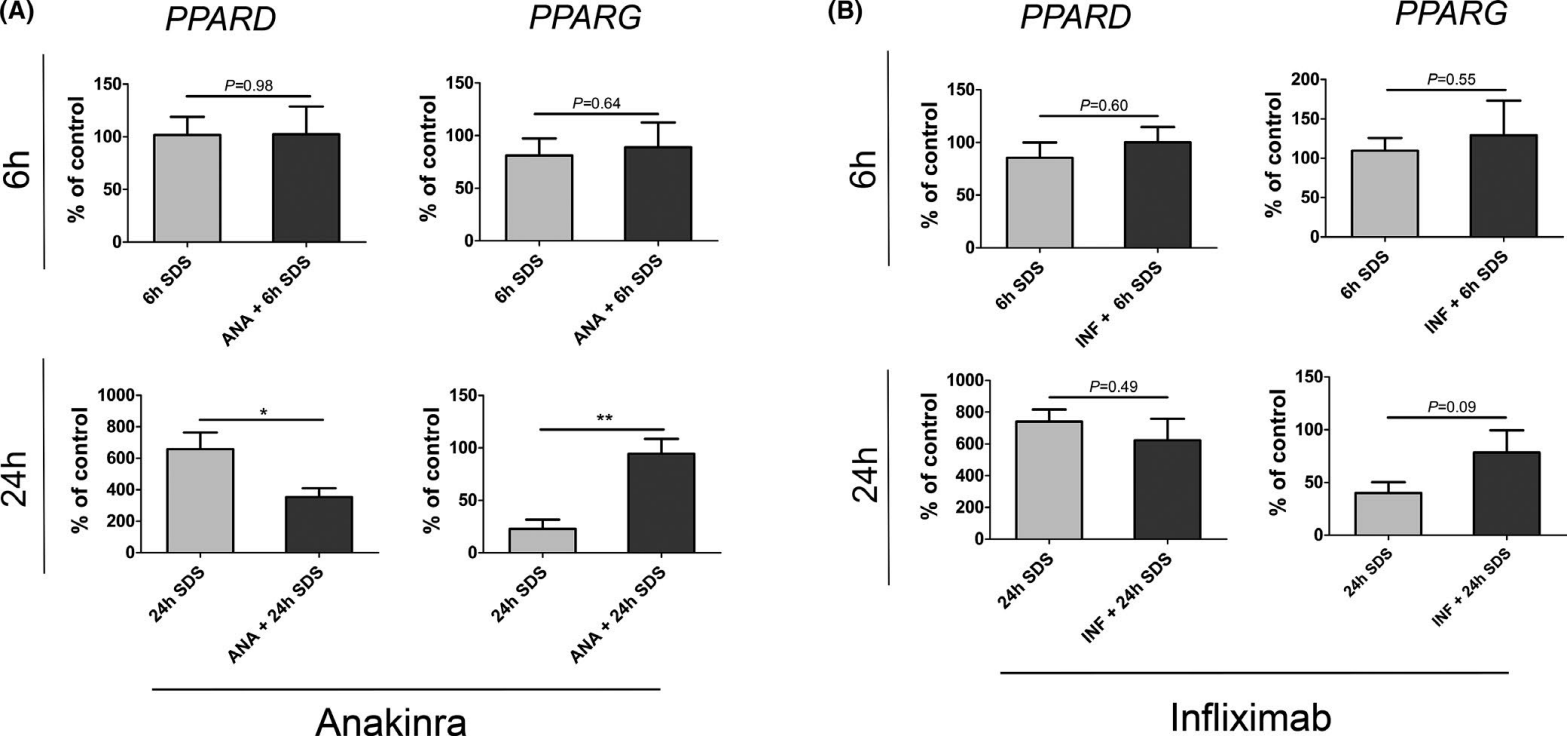
Blocking of IL1 and TNFα signalling in SDS‐mediated barrier impairment in HEEs. (A) HEEs were pretreated with anakinra or saline 24 h and immediately before SDS‐mediated barrier impairment. HEEs were harvested 6 h and 24 h after SDS application. mRNA expression levels of *PPARA, PPARD* and *PPARG* were assessed by RT‐PCR. Combined data from 5 independent, paired experiments are presented as mean ±SEM. Values are given as % of respective ctrl HEEs. As ctrl HEEs were used (1) saline‐pretreated HEEs exposed to PBS for saline‐pretreated HEEs exposed to SDS (groups: “6 h SDS” and “24 h SDS”) and (2) anakinra‐pretreated HEEs exposed to PBS for anakinra‐pretreated HEEs exposed to SDS (groups: “ANA +6 h SDS” and “ANA +24 h SDS”). (B) HEEs were pretreated with infliximab or saline 24 h and immediately before SDS‐mediated barrier impairment. HEEs were harvested 6 h and 24 h after SDS application. mRNA expression levels of *PPARD* and *PPARG* were assessed by RT‐PCR. Combined data from 3 independent, paired experiments are presented as mean ± SEM. Values are given as % of respective ctrl HEEs exposed to PBS. As ctrl HEEs were used (1) saline‐pretreated HEEs exposed to PBS for saline‐pretreated HEEs exposed to SDS (groups: “6 h SDS” and “24 h SDS”) and (2) infliximab‐pretreated HEEs exposed to PBS for infliximab‐pretreated HEEs exposed to SDS (groups: “INF +6 h SDS” and “INF +24 h SDS”). Data were analysed using a paired Student´s t test for all experiments. *p = <0.05; **p = <0.01. IL1, interleukin‐1; TNFα, tumor necrosis factor α; *PPARD*, peroxisome proliferator‐activated receptor β/δ; *PPARG*, peroxisome proliferator‐activated receptor γ

SDS‐ and acetone‐mediated barrier impairment increased *IL1B* and *TNFA* mRNA levels and concomitantly led to a striking decrease of *PPARG* 24 h after barrier perturbation (Figure 2A–C). In line with these findings, treatment of HEEs with IL‐1β and TNF‐α for 24 h clearly reduced *PPARG* gene expression levels (Figure 3C). To test whether abrogation of IL‐1β signalling abolishes the decrease of *PPARG* expression, we treated HEEs with anakinra, a recombinant IL‐1R antagonist. Notably, anakinra treatment abolished SDS‐mediated *PPARG* downregulation at 24 h (Figure 4A). In contrast, blocking of TNFα signalling in HEEs with infliximab, a monoclonal antibody directed towards TNFα, did not abrogate SDS‐induced *PPARG* downregulation (Figure 4B). Taken together, these findings demonstrate that IL‐1β, but not TNF‐α inhibition prevents the downregulation of SDS‐mediated barrier impairment resulting in *PPARG* decrease (Figure 4A). Moreover, blocking of IL‐1R signalling in HEEs by anakinra significantly diminished SDS‐induced *PPARD* upregulation, in contrast to TNF‐α treatment (Figure 4B). Together, these data strongly suggest that IL‐1β, but not TNFα, modulates *PPARG* and *PPARD* expression after SDS‐mediated barrier impairment.

## 4 DISCUSSION

We report two models of acute epidermal barrier perturbation closely mimicking impaired barrier function in human skin. We utilized SDS and acetone to induce barrier impairment. SDS is a detergent that perturbs the cutaneous barrier and has been utilized to instigate irritant contact dermatitis in human skin.^19^, ^33^, ^34^, ^35^, ^36^ Acetone, an organic solvent, has commonly been used to perturb the cutaneous barrier in mice.^29^, ^37^, ^38^, ^39^ Both compounds impair barrier function by removal and/or disturbance of SC intercellular lipid domains that are essential to maintain the epidermal permeability barrier.^19^, ^37^, ^38^, ^39^, ^40^, ^41^, ^42^ As shown in Figure 1C, SDS and, to a lesser extent, acetone decreased TEER, demonstrating that both substances impair barrier function in HEEs with SDS producing a more profound impairment. Our models reliably recapitulate and extent previous data demonstrating upregulation of *IL1A* and *IL1B* and a marked increase of *TNFA* following cutaneous barrier disruption by sequential tape stripping or SDS treatment of normal skin (Figure 2A,B).^34^, ^35^, ^43^, ^44^ Yet, in these prior studies, kinetics of inflammatory mediator mRNA expression was incompletely investigated. Furthermore, whole skin tissue samples, as utilized in most of these reports, do not allow delineating the contribution of the distinct cutaneous compartments and cell types to epidermal cytokine response after epidermal barrier impairment. Thus, changes in gene expression levels, that is in the epidermal compartment might remain undetected.^45^ By focussing on solely the epidermal compartment, we here describe changes that can be exclusively attributed to keratinocytes without interference of immune cells, dermal and subcutaneous tissue. This is of particular interest, since keratinocytes as first line of defense sense danger signals of “barrier impairment” and consequently orchestrate the (inflammatory) response to overcome barrier disturbances.^46^, ^47^ Thus, our models allow investigating the impact of graded barrier impairment on cytokine gene expression, since SDS treatment resulted in a more pronounced barrier perturbation than acetone treatment (Figure 1B,C). Acetone treatment for instance resulted in an increase of *IL1B* expression levels whereas *IL33* mRNA levels were reduced 24 h after barrier impairment. *IL1A* and *TNFA* gene expression was not significantly altered (Figure 2B). Since acetone‐mediated barrier impairment inflicts a less pronounced barrier perturbation, these data demonstrate that *IL1B* and *IL33* are highly sensitive markers of epidermal barrier impairment in human epidermis (Figure 2B).

Furthermore, these findings show that varying degrees of barrier impairment (SDS vs. acetone) lead to distinct cytokine profiles. Intriguingly, *IL33* and *TSLP* mRNA expression was dampened 24 h after SDS and acetone treatment, whereas it was triggered 6 h after SDS treatment only (Figure 2A,B). TSLP and IL33 expression were reported to be increased in human and murine skin as early as 6 h after tape stripping.^43^, ^48^, ^49^, ^50^ These results were acquired in full‐thickness skin samples without investigating temporal kinetics or graded epidermal barrier impairment. By contrast, we document a decrease of *TSLP* and *IL33* gene expression levels following an initial increase after profound epidermal barrier impairment (Figure 2A). Additionally, we report decreased mRNA expression levels of both cytokines 24 h after milder epidermal barrier perturbation, in absence of the earlier (6 h) upregulation (Figure 2B). Both TSLP and IL‐33 were proposed to function as alarmins that alert the immune system and spur a Th2 immune response following tissue damage.^49^, ^51^, ^52^ Moreover, IL‐33 can exert immunosuppressive functions by induction of T‐regs after barrier impairment, thereby preventing exaggerated skin inflammation.^50^ Furthermore, IL‐33 and TSLP were suggested to directly dampen epidermal barrier function by downregulation of *FLG* expression.^53^, ^54^, ^55^ Thus, it is likely that *IL33* and *TSLP* upregulation only occurs shortly after strong epidermal barrier impairment, which requires an involvement of immune cells to protect skin against pathogens or to dampen exaggerated skin inflammation.^50^, ^56^, ^57^ Along these lines, it is tempting to speculate that downregulation of *IL33* and *TSLP* might correspond to the termination phase of epidermal barrier recovery after acute impairment. In this scenario mRNA downregulation of *IL33* and *TSLP* potentially might contribute to epidermal barrier restoration by enhancing expression levels of critical epidermal proteins including *FLG* and *CLDN1*.^54^, ^58^ Moreover, our results show a role of TNF‐α in *IL33* downregulation after epidermal barrier impairment (Figure 3B).^59^ This modulatory function is probably indirect because it requires at least 18 h to take place.

Barrier impairment mediated not only by SDS but also by acetone led to reduced *PPARG* gene expression levels, thereby underscoring the role of PPARγ in epidermal homeostasis (Figure 3C).^26^, ^60^, ^61^, ^62^ These data indicate that PPARγ signalling closely correlates with epidermal barrier fitness. Thus, it is not surprising that PPARγ ligands promote epidermal barrier recovery.^61^ In line, PPARγ activation increases *PPARG* mRNA levels.^26^ These results are in agreement with findings demonstrating reduced *PPARG* expression in inflamed skin lesions ^10^, ^12^, ^14^ and with the beneficial effects of PPARγ ligands in patients with psoriasis and in a murine model of this disease.^4^, ^26^ Moreover, IL‐1β and TNF‐α cytokine treatment decreased *PPARG* gene expression levels (Figure 3C) and abrogation of IL‐1 signalling using anakinra, but not of TNF‐α, restored normal *PPARG* mRNA levels after barrier impairment induced by SDS (Figure 4A). Thus, *PPARG* downregulation might mainly and directly result from upregulation of IL‐1β signalling pathway in keratinocytes. In addition, IL‐1β enhances *PPARD* mRNA levels. This together with the findings that SDS‐mediated barrier impairment induced a much greater increase of *IL1B* gene expression than barrier impairment produced by acetone, implicates that only a profound disturbance of the epidermal barrier triggers *PPARD* mRNA expression via increased IL‐1β levels (Figure 2). In addition, blocking of IL‐1 signalling mitigates the increase of *PPARD* expression levels observed in SDS‐treated HEEs (Figure 4). These data demonstrate that IL‐1β modulates *PPARG* and *PPARD* expression at transcriptional level in human keratinocytes in an autocrine/paracrine manner. Moreover, the here presented data suggest that *PPARA* gene expression might be hardly implicated in epidermal barrier recovery. Furthermore, both in psoriasis and AD *IL1B* gene expression levels are increased.^63^, ^64^ Thus, it is tempting to speculate that IL‐1β contributes to *PPAR* expression changes observed in these diseases.^10^, ^12^, ^14^, ^15^, ^16^, ^17^

In this study, we present an organotypic model to investigate the response of epidermal keratinocytes following barrier perturbation. We demonstrate that IL‐1β and IL‐33 are pertinent markers of epidermal barrier impairment. Furthermore, we show that keratinocyte‐derived IL‐1β modulates *PPARG* and *PPARD* gene expression in human epidermis. In summary, this work may form the basis for future investigations studying the impact of abrogation of IL‐1β signalling on PPARγ and its effects on normalization of barrier homeostasis in common inflammatory skin disorders such as atopic dermatitis and psoriasis.

## CONFLICT OF INTEREST

None.

## AUTHOR CONTRIBUTIONS

SB designed the research study, performed research, analysed data and drafted the manuscript; TK performed research; VMM performed research; RG performed research; MS designed the research, revised the manuscript; SD designed the research, analysed data and revised the manuscript. All authors have read and approved the final version of the manuscript.

## Supporting information

**FIGURE S1.** PPAR expression in IL‐1β‐, TNFα‐ and TSLP‐treated cultured human keratinocytes. Human keratinocytes were grown to a confluency of 70 to 80% and treated with IL‐1β (c: 100 ng/µl), TNFα (c: 10 ng/µl) and TSLP (c: 10 ng/µl) for the periods of time. Thereafter, cells were harvested and subjected to TRIZOL‐based RNA extraction. mRNA expression levels of *PPARA*, *PPARD* and *PPARG* were assessed by RT‐PCR. Combined data from 3 independent experiments are presented as mean ± SEM. Gene expression was normalized to TATA box binding protein and values are presented as fold change vs. PBS treated control keratinocytes. Data were analyzed using a paired Student's t‐test. *p = <0.05; **p = <0.01; ***p = <0.001. IL‐1β, interleukin‐1β; TNFα, tumor necrosis factor α; TSLP, thymic stromal lymphopoietin; *PPARA*, peroxisome proliferator‐activated receptor α; *PPARD*, peroxisome proliferator‐activated receptor β/δ; *PPARG*, peroxisome proliferator‐activated recpetor γ.
