# Supplementary material for: Keratinocyte‐derived IL‐1β induces PPARG downregulation and PPARD upregulation in human reconstructed epidermis following barrier impairment
Source: Exp Dermatol. 2021 Mar 18;30(9):1298–308. doi: 10.1111/exd.14323 (PMC8451818; doi:10.1111/exd.14323)
Supplement: Supplementary file 1 — FIGURE S1. PPAR expression in IL‐1β‐, TNFα‐ and TSLP‐treated cultured human keratinocytes. Human keratinocytes were grown to a confluency of 70 to 80% and treated with IL‐1β (c: 100 ng/µl), TNFα (c: 10 ng/µl) and TSLP (c: 10 ng/µl) for the periods of time. Thereafter, cells were harvested and subjected to TRIZOL‐based RNA extraction. mRNA expression levels of PPARA, PPARD and PPARG were assessed by RT‐PCR. Combined data from 3 independent experiments are presented as mean ± SEM. Gene expression was normalized to TATA box binding protein and values are presented as fold change vs. PBS treated control keratinocytes. Data were analyzed using a paired Student's t‐test. *p = <0.05; **p = <0.01; ***p = <0.001. IL‐1β, interleukin‐1β; TNFα, tumor necrosis factor α; TSLP, thymic stromal lymphopoietin; PPARA, peroxisome proliferator‐activated receptor α; PPARD, peroxisome proliferator‐activated receptor β/δ; PPARG, peroxisome proliferator‐activated recpetor γ. [file EXD-30-1298-s001.docx]

**Supplementary material:**

**Figure S1:**

**
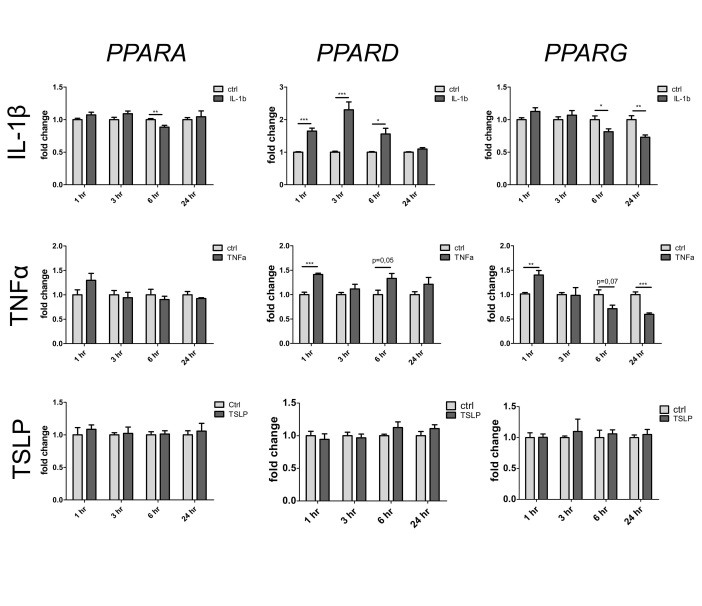
**

**Supplementary Figure S1. PPAR expression in IL-1β-, TNFα- and TSLP-treated cultured human keratinocytes.** Human keratinocytes were grown to a confluency of 70 to 80% and treated with IL-1β (c: 100ng/µl), TNFα (c: 10ng/µl) and TSLP (c: 10ng/µl) for the periods of time. Thereafter, cells were harvested and subjected to TRIZOL-based RNA extraction. mRNA expression levels of *PPARA*, *PPARD* and *PPARG* were assessed by RT-PCR. Combined data from 3 independent experiments are presented as mean ± SEM. Gene expression was normalized to TATA box binding protein and values are presented as fold change vs. PBS treated control keratinocytes. Data were analyzed using a paired Student´s t-test. *p= <0.05; **p= <0.01; ***p= <0.001. IL-1β, interleukin-1β; TNFα, tumor necrosis factor α; TSLP, thymic stromal lymphopoietin; *PPARA*, peroxisome proliferator-activated receptor α; *PPARD*, peroxisome proliferator-activated receptor β/δ; *PPARG*, peroxisome proliferator-activated recpetor γ.
